# The role of object-based attention in semantic working memory

**DOI:** 10.3389/fpsyg.2025.1560745

**Published:** 2025-04-15

**Authors:** Ting Guo, Leyue Ye, Jinfeng Huang, Zhihan Xu

**Affiliations:** ^1^Research Center for Language and Cognition, School of Foreign Languages, Ningbo University of Technology, Ningbo, China; ^2^Guangdong Key Laboratory of Biomedical Measurements and Ultrasound Imaging, School of Biomedical Engineering, Shenzhen University Medical School, Shenzhen, China

**Keywords:** working memory, object-based attention, Duncan task, semantic memory, sentence superiority effect

## Abstract

**Introduction:**

Recent studies suggest that working memory (WM) temporarily stores and processes bindings, while semantically related WM processing interacts with long-term memory (LTM). Semantic information can be categorized into grammatically connected sentences, which benefit from LTM-based semantic and syntactic integration, and isolated word lists, which lack meaningful structural connections. The sentence superiority effect refers to the enhanced memory performance of sentences compared to word lists. This study explores how object-based attention resources contribute to semantic WM processing in second language (L2) learners and examines the impact of object attention task load on sentence superiority.

**Methods:**

We employed the Duncan task as an interference paradigm to investigate whether object-based attention load influences the sentence superiority effect in L2 learners. Participants completed memory tasks involving either isolated word lists or connected sentences while simultaneously performing the interference task.

**Results:**

Findings revealed that connected sentences were more resistant to attention interference compared to word lists, indicating that sentence processing in WM benefits from structural and semantic integration.

**Discussion:**

These results suggest distinct mechanisms of attention resource deployment in semantic processing. The study provides insights into how linguistic context modulates the interaction between attention resources and working memory, highlighting the role of structured language input in cognitive processing.

## 1 Introduction

Building and maintaining a limited set of integrated representations from distinct features (i.e., binding) is a fundamental function of working memory (WM) (Allen et al., 2012; Baddeley et al., 2011). Within this framework, research has established complex interactions between working memory, long-term memory, and attention systems during language processing (Majerus, 2013). Studies using concurrent task paradigms have revealed how attention resources constrain these memory systems’ operations (Barrouillet et al., 2004; Camos et al., 2019), particularly in the context of verbal working memory. However, the specific role of object-based attention in mediating these interactions remains unclear.

Object-based attention, which facilitates the selection and maintenance of integrated object representations, has been extensively studied in visual processing (Chen, 2012; Lin et al., 2021). Research has shown that when attending to a specific object, processing efficiency is enhanced not only for the attended feature but for all features of that object, with attention resources being automatically spread across all features of the selected object (Duncan, 1984; Mack et al., 1992; O'Craven et al., 1999). While the mechanisms of object-based attention have been extensively studied in visual cognition (Wan et al., 2020), its role in semantic working memory remains largely unexplored. The potential role of object-based attention in semantic processing is particularly intriguing because semantic working memory involves complex interactions between temporary representations and long-term knowledge structures (Baddeley et al., 2009).

When investigating these cognitive mechanisms in our current study with second language learners, it is important to consider how such basic cognitive operations manifest across different linguistic contexts. Abutalebi and Green (2007) indicated that second language processing involves additional cognitive demands compared to first language processing. However, other studies have shown that fundamental cognitive mechanisms, including working memory operations and attention resource allocation, function similarly across L1 and L2 processing (Grundy et al., 2017; Wen, 2012). Previous studies have demonstrated that core cognitive processes such as semantic integration and executive control operate on similar principles in both L1 and L2, though they may show different levels of efficiency (Kroll and Bialystok, 2013). This suggests that examining object-based attention effects in L2 semantic processing can provide valuable insights into the fundamental mechanisms of semantic integration, while potentially revealing how these mechanisms adapt to increased cognitive demands.

This is particularly important because semantic working memory involves a unique interaction between temporary representations and long-term knowledge structures (Baddeley et al., 2009). In the current study, we manipulated object-based attention through a dual-task paradigm where participants needed to maintain semantic information while simultaneously processing distracting object representations. This approach allows us to examine how the disruption of object-based attention affects the maintenance and integration of semantic information, providing insights into how attention resources support the binding of semantic elements into coherent representational units in working memory.

One way to examine the role of object-based attention in semantic working memory is by comparing memory performance for connected sentences and unconnected word lists. Connected sentences typically demonstrate superior memory performance compared to unconnected word lists, known as the sentence superiority effect (Potter and Lombardi, 1990). The theoretical foundation for linking object-based attention to sentence processing lies in their shared mechanisms of information integration. Just as object-based attention facilitates the integration of visual features into coherent object representations (Chen, 2012; Duncan, 1984), similar integrative mechanisms appear to operate in sentence processing. Specifically, object-based attention facilitates this integration process by: (1) selecting and maintaining the relationship between sequential words, (2) binding these words into a coherent representational unit, and (3) supporting the activation of relevant semantic schemas from long-term memory (Altmann and Kamide, 2009; Garnham, 1985). The critical role of object-based attention in this process is evidenced by studies showing that disrupting attention resources during sentence processing significantly impairs comprehension and memory performance (Gordon et al., 2001). Furthermore, the sentence superiority effect persists even when controlling for factors such as word frequency and semantic associations (Johnson, 1970; Marks and Miller, 1964), suggesting that the integration benefits arise from higher-level organizational processes that specifically depend on object-based attention resources. Our experimental manipulation of attention through concurrent object processing allows us to directly test how object-based attention constraints affect this integration process.

The relationship between object-based attention and semantic working memory may manifest differently when processing connected sentences versus isolated lexical items. Connected sentences, which can benefit from existing knowledge structures and grammatical rules, might place different demands on object-based attention resources compared to isolated lexical items that lack such organizational support. This integration process in sentence comprehension operates through dynamic interactions between multiple levels, incorporating semantic, syntactic, and contextual information to form coherent representational units (Hagoort, 2005; Pickering and Garrod, 2013). Both sentences and isolated words require object-based attention, but to different extents. For sentences, object-based attention operates in interaction with long-term memory semantic, syntactic, and contextual processes, which reduces the explicit allocation of attentional resources. In contrast, isolated lexical items require more explicit allocation of object-based attention resources, as they lack the supportive long-term memory processes and inherent organizational structure that sentences provide. This makes object-based attention for isolated words more vulnerable to disruption.

Research using concurrent task paradigms has demonstrated that attention demands can significantly impact working memory maintenance and processing (Barrouillet et al., 2004; Camos et al., 2019). Object-based attention resources are required for both the verbal memory task and the secondary visual task, creating a competition for limited attentional resources. When secondary task demands increase, attention is necessarily drawn away from the memoranda, affecting performance. By manipulating this attention load, we can directly test whether sentence processing is more resistant to attention interference compared to isolated word processing. Given these theoretical considerations about the differential roles of object-based attention (Chen, 2012; Duncan, 1984; Gao et al., 2022; Jeong and Cho, 2024) in processing connected and isolated verbal materials, and the established interaction between language processing and working memory systems (Majerus, 2013), a direct empirical investigation is needed to examine how varying levels of object-based attention load might differentially affect the processing of sentences versus isolated words. We hypothesize that if sentence processing indeed relies more on automatic binding mechanisms, it should be more resistant to object-based attention interference compared to the processing of isolated words.

To investigate how object-based attention influences semantic working memory processing, we employed the Duncan task, which specifically taxes object-based attention resources. This dual-task approach aligns with established paradigms for investigating attention-memory interactions (Barrouillet et al., 2004), while specifically focusing on object-based attention demands. By manipulating object-based attention load through this task while participants process semantic information, we aim to elucidate the role of object-based attention in semantic working memory and its potential interaction with different types of semantic materials.

## 2 Methods

In Baddeley et al.’s study, three different types of semantic information were used: natural language sentences, semantically constrained sentences (CS), and constrained word lists (CL) (Baddeley et al., 2009). The working memory processing efficiency of the two types of sentences is better than that of word lists. However, in subsequent experiments, only CS and CL were used, as the inclusion of natural sentences led to substantial variability in performance. This variability stemmed from differences in sequence length, semantic content, and additional contributions from episodic LTM.

Building on previous studies, the current study also focused on CS and CL conditions in our current study. This design was made to avoid the variability introduced by natural sentences, which could complicate the analysis due to differences in sequence length, semantic content, and additional contributions from episodic LTM. By using only these two types of materials, we aimed to create a more controlled and consistent environment to examine the effects of semantic chunking and working memory load on sentence recall.

### 2.1 Evaluation of experimental materials

#### 2.1.1 Participants

Fifteen paid student volunteers from Ningbo University of Technology (10 females; age range: 18–21 years; mean = 19.13 years, SD = 1.06) participated in the pre-test. All participants had scored above 500 (range: 504–560; mean = 530.21) on the College English Test Band 4 (CET-4), reported normal or corrected-to-normal vision, and provided written informed consent. This proficiency threshold was established to ensure participants could effectively process the English experimental materials while avoiding ceiling effects that might occur with native English speakers. The study protocol was approved by the university’s Institutional Review Board.

#### 2.1.2 Apparatus and materials

The experiment was programmed in MATLAB 2021a using Psychophysics Toolbox Version 3 (PTB-3) (Brainard, 1997) and presented on a 24-inch LCD monitor (1920 × 1,080 pixels, 60 Hz). The stimuli were presented against a gray (128, 128, 128; RGB) background.

Two types of sequences were developed: CS and CL conditions. Following Baddeley et al. (2009), we adopted their established word pool, which contained words categorized into nouns (e.g., “Peter,” “pilot,” “car”), adjectives (e.g., “red,” “large,” “wealthy”), verbs (e.g., “gave,” “cleaned”), and adverbs (e.g., “rapidly,” “easily”). These words were originally selected from Kučera and Francis (1967) corpus for their medium-high frequency (mean frequency = 199.55) and were validated in the study of Baddeley et al. (2009). All stimuli were generated using text-to-speech (TTS) synthesis technology and presented individually to eliminate prosodic and co-articulatory cues.

For CS conditions, sequences were constructed by selecting 1–2 words from each noun and adjective subset, one verb, and one adverb without replacement. Function words (e.g., “the,” “to,” “and,” “from,” “for”) were included to create grammatically correct sentences but excluded from the analysis. For example, a CS sequence might be “The wealthy pilot rapidly gave the red car.” CL conditions were then derived by removing function words from these sentences, with word order manipulated to minimize grammatical structure and semantic relationships (e.g., “pilot red rapidly wealthy gave”).

Given that these materials were originally designed for native English speakers, our initial testing with non-native participants revealed a floor effect. To address this challenge, we modified the sequences by reducing their length while maintaining the same word pool and construction principles.

#### 2.1.3 Design and procedure

After these modifications, participants completed oral recall tasks for both conditions.

Each participant was tested in a single session lasting approximately 25 min. At the start of the experiment, instructions were displayed, and participants pressed the spacebar to begin once they understood the task. Following the keypress, a fixation cross appeared on the screen, and auditory stimuli were presented through a pair of over-ear headphones with an integrated microphone. The audio was played at a rate of one word per second for both the CL and CS conditions, and the same digital sound files were used for both materials. After the audio sequence finished, a new set of instructions appeared, asking participants to orally recall the items in the order they had heard them. After recalling, participants pressed a key to proceed to the next trial. This sequence continued until all trials were completed.

Each experimental block included four practice trials, followed by 16 test trials: 16 CL and 16 CS sequences. Participants with odd-numbered subject numbers started with the constrained lists, while participants with even-numbered subject numbers started with the sentences.

For the recall scoring, an item was considered correct if it was recalled in its proper relative position to other items in the sequence. This scoring approach was chosen as it captures how sequential information is maintained in working memory, where items are typically encoded in relation to their neighbors rather than as independent units. The position of the first and last items in the sequence were treated as exceptions, where they were only scored as correct if they appeared in the same positions during recall. For example, if the sequence “ABCDE” was recalled as “CBADE,” only the last two items (D and E) would be considered correct because the relative order of C and B had been reversed. In contrast, if the sequence was recalled as “BCADE,” all words except for A would be counted as correct, as the relative order of the remaining words (B, C, D, and E) had been maintained. In trials where participants failed to recall any correct words, their performance was recorded as zero points. This scoring method is consistent with the procedure outlined in previous research (Baddeley et al., 2009).

The scores were calculated and compared with previous studies, as presented in Table 1.

**Table 1:** The results of material assessment in word length and recall scores.

		Numbers of words		Score	
	Participants	Constrained lists	Constrained sentences	Constrained lists	Constrained sentences
Baddeley et al. (2009)	Native speaker	6 words	8 words	5.18 (1.22)	7.20 (1.04)
Current study	English learners	5 words	6 words	2.53 (1.34)	3.73 (1.69)

As shown in the Table 1, although English learners’ recall scores were lower than native speakers, they demonstrated measurable performance without floor effects. Given that participants could complete the modified materials while maintaining sufficient cognitive demands, we proceeded with these adjusted materials in our main experiment.

### 2.2 Main experiment

#### 2.2.1 Participants

An *a priori* power analysis using G*Power 3.1 (Faul et al., 2007; Susanne et al., 2007) suggested a sample size of *N* = 28 for a 2 × 2 repeated-measures analysis of variance (ANOVA) with a moderate effect size of *f* = 0.25, α = 0.05 and 1-β = 0.80. We adopted a sample size of *N* = 28 to obtain the desired sample size.

Twenty-eight paid student volunteers from the Ningbo University of Technology participated in our study (14 females; age range: 18–21 years; mean = 19.25 years, SD = 1.14). All participants’ English proficiency was assessed using the CET- 4 (range: 500–589, mean = 548.86). In addition, all participants reported normal or corrected-to-normal visual acuity and provided written informed consent before participating in the experiment. The Institutional Review Board of Ningbo University of Technology approved the experimental protocols.

#### 2.2.2 Apparatus and materials

In the main experiment, we used the modified materials that had been assessed in the preliminary evaluation (see Table 1), consisting of 16 sets of CL and CS sequences. All apparatus used for stimulus presentation and scoring methods remained identical to those in the preliminary experiment. A delayed-match task paradigm was employed, in which participants performed a secondary task between the presentation and recall of sequences. The secondary task used the Duncan task (Duncan, 1984), with parameters based on Zhou et al. (2021).

#### 2.2.3 The Duncan task

The Duncan task (Duncan, 1984) is a widely used paradigm to assess object-based attention. In this task, participants need to process multiple features of overlapping objects, which requires the allocation of object-based attention resources. We chose this task as our secondary task because it specifically targets object-based attention processes without substantially engaging verbal working memory resources.

In our implementation, following Zhou et al. (2021), the stimulus consisted of two superimposed black objects—a box and a line. The box (1.02° width) was either short (1.02°) or tall (1.43°) and had a gap (0.31° width) on either the left or right side. The line (3.19° long) was either dashed or dotted and tilted 8° to either the left or right side. A backward mask (2.11° width × 2.86° height) was used following the presentation of the stimuli. Participants were required to make two consecutive judgments about the box features: first identifying the gap location, then judging the box height. This dual-judgment requirement ensures active engagement of object-based attention resources.

#### 2.2.4 Design and procedure

The main experiment employed a 2 (memory condition: CL vs. CS) × 2 (task load: no Duncan task vs. with Duncan task) within-subjects design. The Duncan task was used to manipulate object-based attention load, allowing us to examine how different types of verbal materials respond to attention interference. Specifically, by comparing performance between conditions with and without the Duncan task, we could assess the differential effects of attention load on sentence versus word list processing. This design followed the procedures used in previous research (Baddeley et al., 2009; Shen et al., 2013).

At the beginning of the experiment, participants completed five practice trials to familiarize themselves with the task requirements. After this practice phase, participants proceeded to the main experiment.

Each trial began with the presentation of a fixation cross on the screen for 500 ms, followed by the auditory stimulus: either a constrained word list or a constrained sentence, depending on the condition. Participants were instructed to listen carefully and remember the content of the auditory material.

In the secondary-task conditions (with Duncan task), after the auditory presentation, a box with an oblique line appeared on the screen. Participants were required to make two judgments regarding the box: first, to determine whether the gap was on the left or right side; and second, to judge the box height (low or high). Participants were given examples of “low” and “high” boxes before the experiment to ensure they understood the task. The gap direction was indicated by the left or right side of the box, and the height was represented by the distance from the top or bottom of the box. A visual mask appeared on the screen immediately after the box, followed by two questions. The first question asked participants to indicate the direction of the gap (press F for left or J for right), and the second question asked them to judge the height of the box (press F for low or J for high). Participants were required to respond within a 2,000-ms time window. In the no-Duncan-task condition, participants were instructed to press the spacebar to ignore the secondary task stimuli and focus only on the memory task. If participants failed to respond within the time limit or made an incorrect response, the secondary task in that trial was considered incorrect.

After completing the Duncan task (or pressing spacebar in the no-Duncan-task condition), participants were prompted to verbally recall all the words they remembered from the current trial. Participants’ verbal responses were recorded through a headset microphone for each trial. The experimental program (implemented in Matlab Psychophysics Toolbox) simultaneously recorded both the audio responses and generated a trial-by-trial record of presented stimuli sequence.

**Figure 1:**
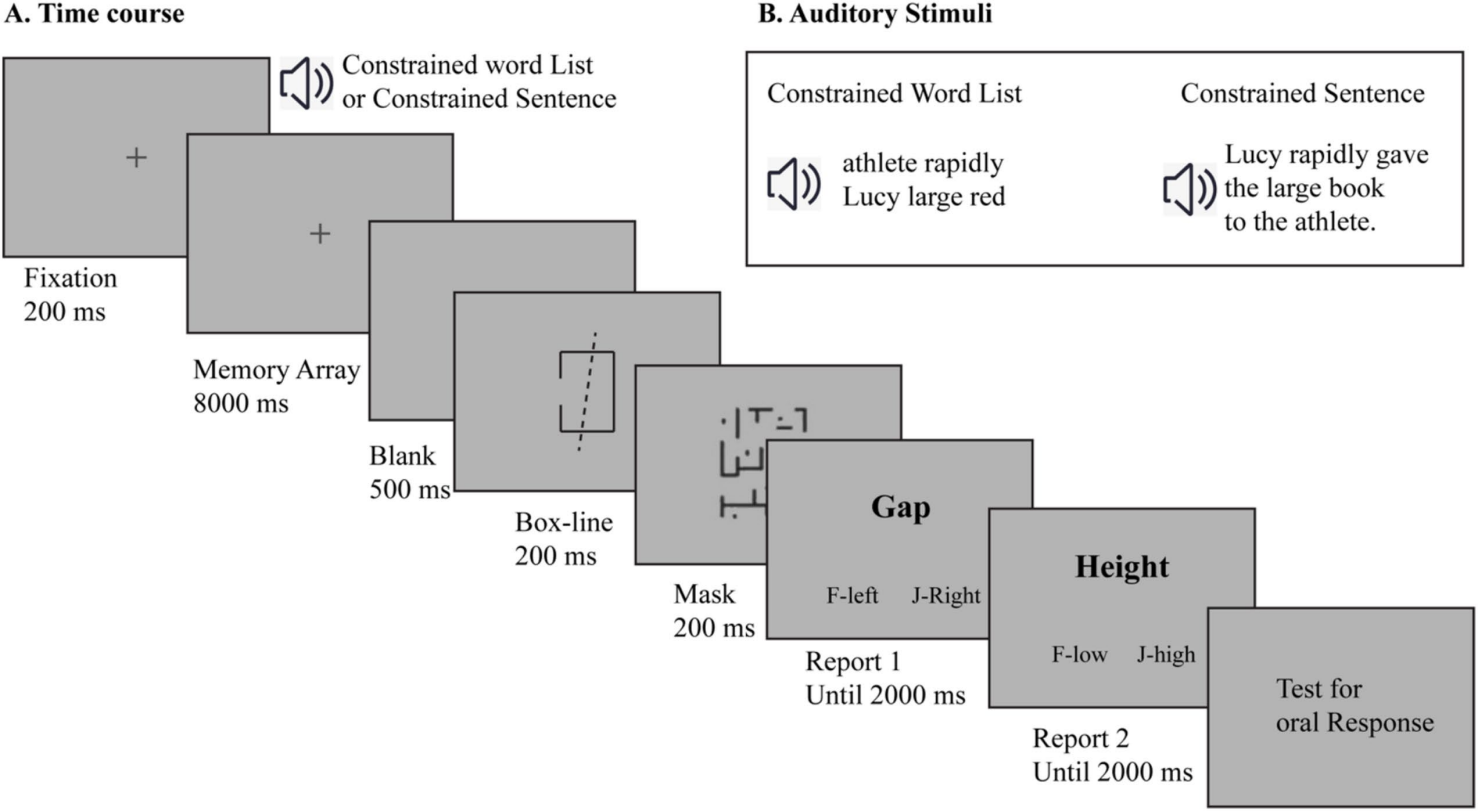
Time course **(A)** and sample stimuli **(B)** of the main experiment.

Once they complete the recall task, participants can press the spacebar at any time to proceed to the next trial. The procedure for the main experiment is presented in Figure 1.

### 2.3 Data analysis

The scoring procedure followed the criteria established by Baddeley et al. (2009). Each participant’s trial-specific audio recordings were scored post-experiment, with the corresponding stimulus sequence records serving as reference. To ensure scoring reliability, all responses underwent a double-check procedure where scores were verified against both the audio recordings and the stimulus sequence records.

To ensure measurement equivalence across conditions while preserving the relative performance differences between groups, participants’ raw scores were standardized using a percentage transformation. Specifically, raw scores were divided by the maximum possible score for each condition (5 points for CL and 6 points for CS) and then multiplied by 100 to obtain percentage scores (Tabachnick et al., 2013). Additionally, task cost was calculated to assess the impact of the Duncan task on memory performance, specifically examining how the presence of the Duncan task influenced the memory effects of different types of semantic materials (CL vs. CS). Furthermore, accuracy scores for the Duncan task were recorded and used as a reference for further analyses. A two-way repeated measures ANOVA was conducted to assess the main and interaction effects of memory condition (CL vs. CS) and task load (with Duncan task vs. no Duncan task) as within-subjects factors. The *η*^2^ was calculated as an estimate of effect size. To examine the difference in cognitive load between CL and CS conditions, we compared the disruption of the Duncan task between conditions. Specifically, we calculated the cost of the secondary task by subtracting the memory performance under the Duncan task condition from that under the no-Duncan task condition. Following previous studies (Gao et al., 2017), we conducted an independent sample *t*-test to compare the disruptions of the Duncan task between CL and CS conditions.

## 3 Results

**Figure 2:**
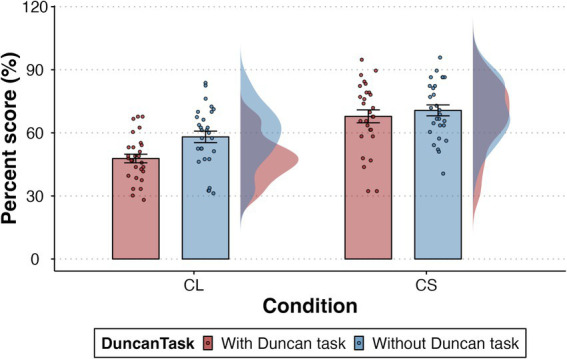
Mean percentage scores of semantic WM performance across conditions. Data distributions are shown as violin plots, with individual data points overlaid. Error bars represent ± SE.

The mean percentage scores for the semantic WM task in each condition are presented in Figure 2, which shows the differences in memory performance across conditions. The detailed descriptive statistics are provided in Table 2, with means and stand errors. The results of ANOVA showed a significant main effect of memory condition, *F*(1, 27) = 64.54, *p* < 0.001, *η*^2^ = 0.262. This indicates that participants performed significantly better in the CS condition compared to the CL condition. Similarly, a significant main effect of task load was found, *F*(1, 27) = 11.43, *p* = 0.002, *η*^2^ = 0.054. This suggests that the presence of the Duncan task had a significant impact on memory performance. Furthermore, the interaction between memory condition and task load was also significant, *F*(1, 27) = 7.92, *p* = 0.009, *η*^2^ = 0.018, indicating that the effect of task load on memory performance differed between the CL and CS conditions.

**Table 2:** Descriptive statistics for memory performance across experimental conditions.

Condition	Task	Task accuracy	Mean percentage scores
CL	With Duncan task	86.94 (1.12)%	47.8 (2.05) %
	No Duncan task	–	60.52 (2.73) %
CS	With Duncan task	86.38 (1.13)%	67.85 (3.07) %
	No Duncan task	–	70.68 (2.59) %

To explore the significant interaction effect, pairwise comparisons using the Bonferroni adjustment were conducted. The results are presented as follows: A significant difference was found between the CS condition and the CL condition, both with and without the Duncan task (*t* = −7.91, *p* < 0.001; *t* = −4.97, *p* < 0.001). These results indicate that participants in the CS condition performed significantly better than those in the CL condition. A significant difference was also observed between the CL condition with and without the Duncan task (*t* = −4.05, *p* < 0.001), suggesting that the load of the Duncan task resulted in decreased performance in the CL condition. However, no significant effect of the Duncan task on memory performance was observed in the CS condition.

**Figure 3:**
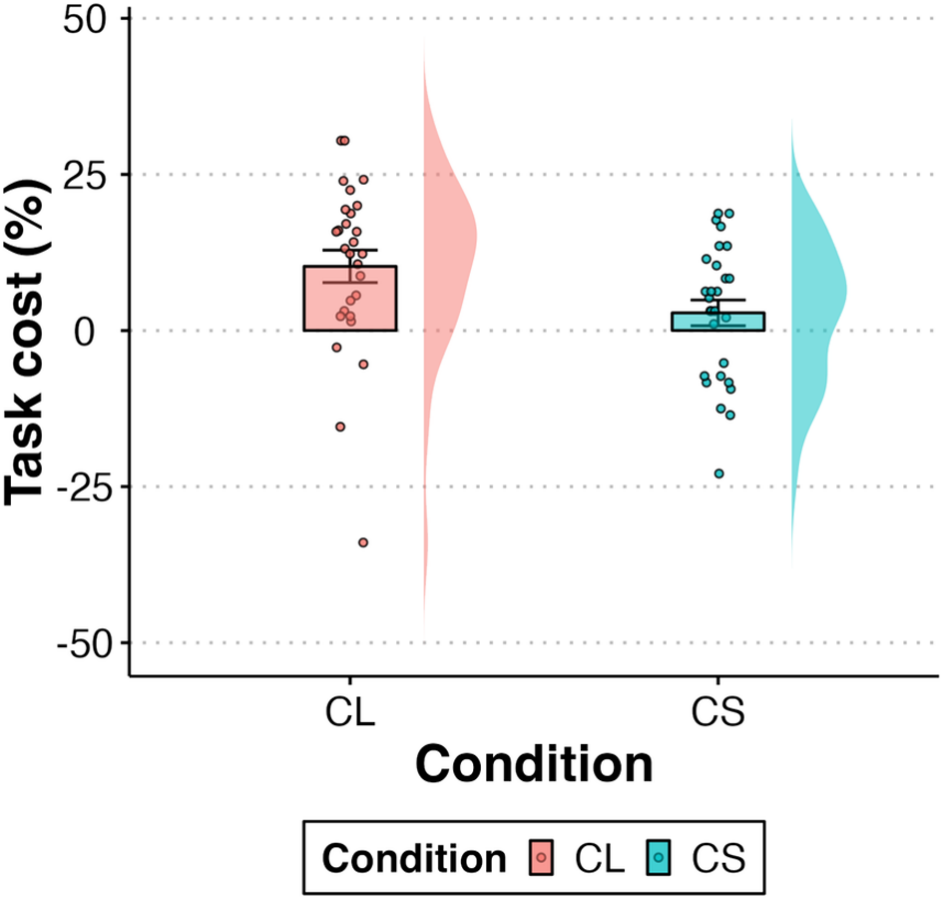
Task cost (performance decline due to Duncan task interference) in different conditions. Data distributions are shown as violin plots, with individual data points overlaid. Error bars represent ± SE.

The cost of Duncan task were presented in Figure 3, the results indicated that the cost of Duncan task was significantly higher in the CL condition than in the CS condition (*t* = 2.24, *p* = 0.03).

## 4 Discussion

The present study investigated how object-based attention affects semantic working memory performance in different linguistic contexts (CL vs. CS). Using the Duncan task as a secondary task to manipulate object-based attention load, we found that semantic working memory performance was significantly affected by attention resources, which is consistent with previous findings on working memory processing. Furthermore, the effect of attention load on semantic processing was modulated by linguistic context. Memory performance for CL condition was more susceptible to interference from the concurrent Duncan task compared to CS condition, as evidenced by the higher task cost in the CL condition. This suggests that connected sentences, which can utilize grammar chunking and establish meaningful associations with long-term memory, require fewer object-based attention resources. However, when processing individual lexical items without semantic connections, participants relied more heavily on object-based attention resources, making their performance more vulnerable to interference from the attention-demanding secondary task. These findings provide new insights into the role of object-based attention in semantic working memory processing and demonstrate that the sentence superiority effect may be partially attributed to more efficient attention resource deployment during semantic encoding.

### 4.1 The sentence superiority effect in memory performance

Firstly, we found significant sentence superiority effects in the current study, where the memory performance of CS sequences was significantly higher than CL sequences. This robust effect demonstrates that the organizational structure of sentences substantially enhances working memory capacity beyond the typical limitations observed with isolated lexical items. Such enhancement reflects not only the basic chunking mechanisms but also the efficient binding of semantic and syntactic information into coherent representational units (Jefferies et al., 2004). This binding process appears to be facilitated by automatic integration mechanisms that operate at multiple linguistic levels (Baddeley et al., 2011).

The observed sentence superiority effect aligns with previous research on working memory binding, where structured linguistic input has been shown to dramatically improve memory performance compared to random word sequences (Treisman and Zhang, 2006). This advantage reflects the fundamental ability of the human memory system to leverage pre-existing knowledge of language patterns and grammatical rules, creating stable object representations that can be maintained with relatively less attention resources (Luck and Vogel, 2013). Importantly, this superiority effect remained stable even under varying attention conditions, suggesting that the advantage of sentential context represents a fundamental characteristic of semantic memory processing rather than a mere strategy-dependent phenomenon. The consistent performance advantage in the CS condition indicates that sentence-level processing may operate through relatively automated binding mechanisms that have been established through extensive exposure to language patterns (Treisman and Zhang, 2006). This automatic binding process appears to be particularly efficient in integrating multiple pieces of information into unified object representations, consistent with theories of object-based attention in visual processing (Chen, 2012; Scholl, 2001).

### 4.2 Differential effects of object-based attention load

The interaction between memory condition and task load revealed distinct patterns of attention resource dependency across linguistic contexts. The Duncan task, which specifically targeted object-based attention, demonstrated asymmetric interference effects on semantic processing: while significantly disrupting performance in the CL condition (*t* = −4.05, *p* < 0.001), it showed minimal impact on CS performance. This differential sensitivity to attention interference aligns with previous research on object-based attention in visual cognition (Duncan, 1984; Scholl, 2001), suggesting similar principles in semantic processing.

The selective interference observed in the CL condition indicates that processing individual lexical items relies heavily on object-based attention resources, similar to how attention operates on individual objects in visual processing (Chen, 2012). When these resources are depleted by a concurrent task, the ability to maintain and process isolated lexical items is significantly compromised. In contrast, sentence processing in the CS condition demonstrated remarkable resilience to attention interference, suggesting that connected sentences engage more efficient processing mechanisms through existing syntactic and semantic frameworks (Jefferies et al., 2004).

These findings extend our understanding of attention-memory interactions by demonstrating that the deployment of object-based attention resources in semantic working memory is modulated by linguistic context. The differential resource requirements between CS and CL conditions suggest that the cognitive system can flexibly adapt its processing mechanisms based on available linguistic structure.

### 4.3 Semantic integration with long-term memory

The distinct patterns of attention resource dependency between CS and CL conditions can be explained through the mechanisms of semantic chunking and long-term memory integration. In the CS condition, the relative immunity to attention interference, evidenced by both the interaction effect (*F*(1, 27) = 7.92, *p* = 0.009) and lower task cost, suggests that sentences are processed through qualitatively different mechanisms than individual lexical items (Jefferies et al., 2004).

When processing connected sentences, the cognitive system appears to capitalize on existing linguistic knowledge stored in long-term memory. This allows for rapid integration of individual words into meaningful units through semantic chunking, effectively reducing the processing load on working memory. The established syntactic and semantic frameworks in long-term memory provide ready-made templates for organizing incoming information, thereby reducing the demand for object-based attention resources during encoding and maintenance. In contrast, the processing of isolated lexical items in the CL condition cannot benefit from such integrative mechanisms. Without meaningful connections between items or support from existing knowledge structures, each word must be maintained as a separate entity in working memory, placing greater demands on object-based attention resources. This explains the higher susceptibility to interference from the Duncan task in the CL condition, as maintaining distinct, unconnected items relies more heavily on attention-dependent working memory processes.

### 4.4 Integration with working memory models

Our findings can be meaningfully interpreted within the framework of established working memory models, particularly Baddeley’s multicomponent model. The differential attention requirements between CS and CL conditions align with the role of the episodic buffer, which serves as an interface between working memory and long-term memory systems (Baddeley, 2000). The reduced attention dependency we observed in sentence processing suggests that when linguistic input is structured, the episodic buffer can more efficiently bind information through existing language schemas, requiring fewer object-based attention resources. This interpretation is consistent with recent research demonstrating how prior knowledge facilitates working memory processing through more efficient binding mechanisms (Thalmann et al., 2019).

Furthermore, our results extend theoretical understanding of how object-based attention interfaces with semantic processing in working memory. The selective interference pattern we observed supports Cowan’s embedded processes model (Cowan, 2017), which emphasizes the role of attention in maintaining activated representations. Specifically, our findings suggest that object-based attention resources play a crucial role in maintaining individual lexical items as distinct entities in working memory, while sentence-level processing benefits from automated binding mechanisms established through linguistic experience (Majerus, 2019). This differential engagement of attention resources provides new insights into how semantic information is organized and maintained within working memory, suggesting that the hierarchical structure of language can fundamentally alter how attention resources are deployed during semantic processing.

### 4.5 Second language processing and working memory

Henrich et al. (2010) emphasized the importance of examining cognitive processes across diverse linguistic populations. The current study explores how semantic working memory functions in non-native language processing, particularly in L2 learners. L2 learners in our study showed notable cognitive demands when processing isolated words (CL condition). However, the structured nature of sentences (CS condition) significantly reduced these demands. L2 learners exhibited a clear sentence superiority effect, with sentence processing requiring fewer attention resources than word lists. This pattern of results suggests that mechanisms like syntactic chunking and semantic integration remain effective in L2 contexts, even under increased cognitive load (Kroll and Bialystok, 2013). In summary, these findings demonstrate that L2 learners can optimize cognitive resource allocation through linguistic structure, maintaining performance even under cognitive load. These results suggest that leveraging sentence-level processing may enhance cognitive efficiency in L2 learning and instruction.

### 4.6 Limitations and future study

Several limitations of the present study should be noted. First, while our results demonstrate how L2 learners utilize sentence structure and deploy attention resources in English verbal working memory tasks, future studies could directly compare L1 and L2 groups to examine potential differences in attention resource allocation and semantic integration strategies between native and non-native speakers.

Second, while our study used the Duncan task to manipulate object-based attention load, future research could employ different types of attention tasks to examine whether the observed effects are specific to object-based attention or generalize to other attention mechanisms. This would provide a more comprehensive understanding of how different aspects of attention interact with semantic processing in working memory.

Third, our study focused on relatively simple sentences and word lists. Future research could systematically vary the complexity of linguistic materials to investigate how syntactic complexity and semantic richness might modulate the relationship between attention resources and working memory processing. This could help clarify whether the reduced attention dependency we observed in sentence processing extends to more complex linguistic structures.

Finally, future studies could explore how individual differences in L2 proficiency and working memory capacity influence the ability to leverage linguistic structure for memory enhancement. This could provide insights into the relationship between language expertise and cognitive resource management in bilingual processing.

## 5 Summary

In conclusion, the present study demonstrated that object-based attention resources play a crucial role in semantic working memory processing, with their impact being modulated by linguistic context. Our findings revealed a robust sentence superiority effect in L2 learners, indicating that connected sentences are processed more efficiently than lexical lists. Critically, the differential interference patterns observed under the Duncan task suggest that sentence processing relies less on object-based attention resources, likely due to effective semantic chunking and integration with long-term memory. These findings provide new insights into how attention resources are deployed during semantic processing in working memory and extend our understanding of the cognitive mechanisms underlying the sentence superiority effect in second language processing.

## Data Availability

The datasets presented in this study can be found in online repositories. The names of the repository/repositories and accession number(s) can be found below: The datasets for this study can be found in the [Open Science Framework] [https://osf.io/v97yc/].
